# Supplementary material for: Calcium-Dependent Protein Kinase GhCDPK16 Exerts a Positive Regulatory Role in Enhancing Drought Tolerance in Cotton
Source: Int J Mol Sci. 2024 Jul 30;25(15):8308. doi: 10.3390/ijms25158308 (PMC11311755; doi:10.3390/ijms25158308)
Supplement: Supplementary file 1 [file ijms-25-08308-s001.zip › Supplementary Figures.pdf]

Supplementary Figures for

Calcium-dependent protein kinase GhCDPK16 exerts a positive regulatory role in enhancing drought tolerance in cotton

Figure S1. Amino acid sequence alignment of GhCDPK16 with AtCDPK4 and AtCDPK11.

Figure S2. Expression levels of abiotic stress-related genes in *GhCDPK16* transgenic lines.

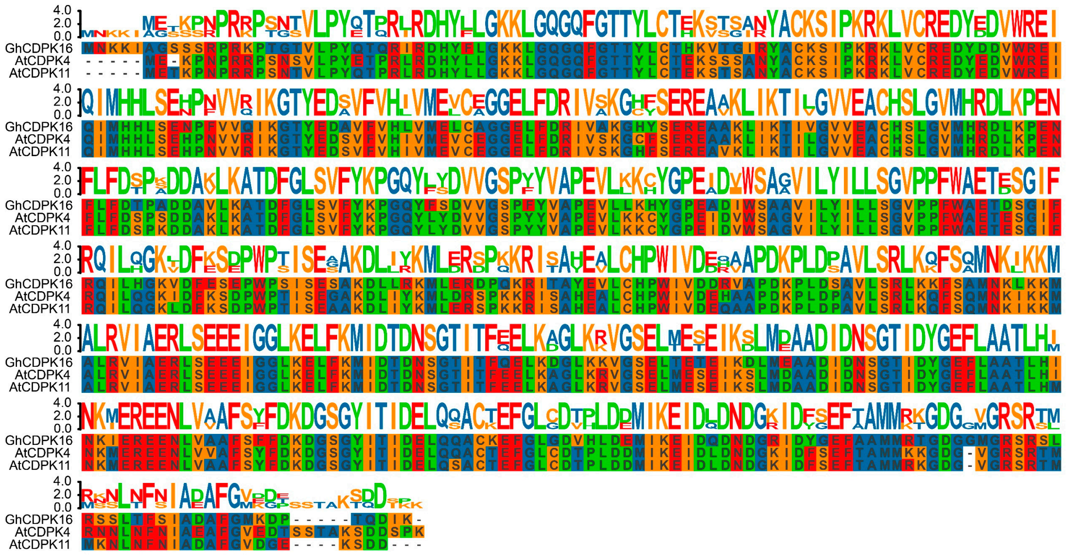

Figure S1. Amino acid sequence alignment of GhCDPK16 with AtCDPK4 and AtCDPK11. The protein sequences of GhCDPK16, AtCDPK4 and AtCDPK11 shared high similarity. Amino acid sequences were aligned using the BioEdit Software via the Clustal W method.

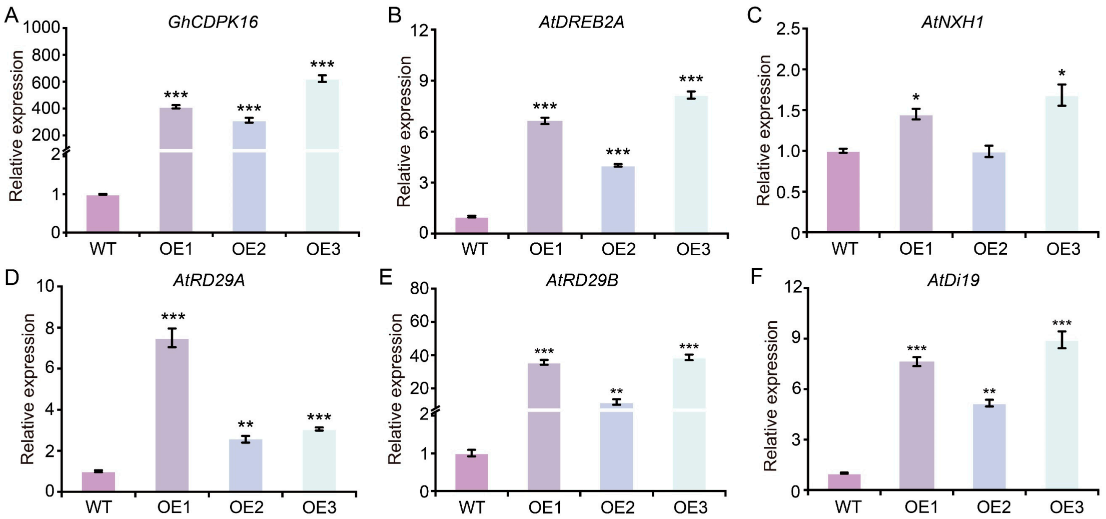

Figure S2. Expression levels of abiotic stress-related genes in *GhCDPK16* transgenic lines **(A)** The expression level of *GhCDPK60* in overexpressed Arabidopsis. **(B-F)** Relative expression levels of several stress-related genes, including *AtDREB2A* **(B)**, *AtNXH1* **(C)**, *AtRD29A* **(D)**, *AtRD29B* **(E)**, and *AtDi19* **(F)** in WT and *GhCDPK16*-overexpressing plants after drought treatment. OE1-OE3 represented independent homozygous Arabidopsis lines overexpressing *GhCDPK16*. Values represented the mean ± SD from three biological replicates. $*P < 0.05$, $**P < 0.01$ and $***P < 0.001$ by Student's *t* test.
