# Supplementary material for: Calcium-Dependent Protein Kinase GhCDPK16 Exerts a Positive Regulatory Role in Enhancing Drought Tolerance in Cotton
Source: Int J Mol Sci. 2024 Jul 30;25(15):8308. doi: 10.3390/ijms25158308 (PMC11311755; doi:10.3390/ijms25158308)
Supplement: Supplementary file 1 [file ijms-25-08308-s001.zip › Supplementary Tables.pdf]

Supplementary Tables for

Calcium-dependent protein kinase GhCDPK16 exerts a positive regulatory role in enhancing drought tolerance in cotton

Table S1. Analysis of *GhCDPK16* promoter.

Table S2. Primer pairs used in this study.

Table S1 Analysis of *GhCDPK16* promoter

Element	Sequence	Number	Function	Type
ACE	CTAACGTATT	1	cis-acting element involved in light responsiveness	Plant growth and development
Box 4	ATTAAT	4	part of a conserved DNA module involved in light responsiveness	Plant growth and development
GA-motif	ATAGATAA	1	part of a light responsive element	Plant growth and development
GATA-motif	AAGGATAAGG	1	part of a light responsive element	Plant growth and development
G-Box	CACG	2	cis-acting regulatory element involved in light responsiveness	Plant growth and development
ABRE	ACGTG	1	cis-acting element involved in the abscisic acid responsiveness	Phytohormone responsive
ERE	ATTTTAAA	6	cis-acting element involved in the ethylene responsiveness	Phytohormone responsive
MYB	AACCA	7	MYB binding sites involved in drought induction	Abiotic and biotic stresses
MYC	CAATTG	1	MYC binding sites involved in drought induction	Abiotic and biotic stresses
STRE	AGGGG	1	stress-response element	Abiotic and biotic stresses
WUN-motif	AAATTTCCT	1	wound-responsive element	Abiotic and biotic stresses

Table S2 Primer pairs used in this study

Usage	Primer Name	Sequence (5′ - 3′)
Construct for overexpression	pBI121-GhCDPK16-F	AGAACACGGGGGACTCTAGAATGAATAAGAAAATTGCAGG
	pBI121-GhCDPK16-R	GATCGGGGAAATTCGAGCTCTCATTTAATATCCTGTGTTGGGT
Construct for localization analysis	1305-35S-GhCDPK16-GFP-F	GCCCAGATCAACTAGTATGAATAAGAAAATTGCAGG
	1305-35S-GhCDPK16-GFP-R	TCGAGACGTCTCTAGATTTAATATCCTGTGTTGGGT
	1305-35S-GFP- GhCDPK16-F	GACGAGCTGTACAGATCTATGAATAAGAAAATTGCAGG
	1305-35S-GFP- GhCDPK16-R	GCGGCCGCTTTAAGATCTTCATTTAATATCCTGTGTTGGGT
Construct for VIGS analysis	TRV2:GhCDPK16-F	TCTGTGAGTAAGGTTACCGAATTCCCTTGCTGCTACACTGCACA
	TRV2:GhCDPK16-R	CCATGGAGGCCTTCTAGAGAATTCCATACCCCCATCACCCGTTC
RT-qPCR	qRT-GhCDPK16-F	CAAGAAAGCCCACAGGTACGGT
	qRT-GhCDPK16-R	GGTGGTGCCAAATTGACCTTGC
	qRT-GhACT4-F	TTGCAGACCGTATGAGCAAG
	qRT-GhACT4-R	ATCCTCCGATCCAGACACTG
	qRT-GhUBQ7-F	GAATGTGGCGCCGGGACCTTC
	qRT-GhUBQ7-R	ACTCAATCCCCACCAGCCTTCTGG
	qRT-AtSAND-qF	AACTCTATGCAGCATTTGATCCACT
	qRT-AtSAND-qR	TGATTGCATATCTTTATCGCCATC
	qRT-AtNXH1-F	ACCCCAAAATCCATACATATCCC
	qRT-AtNXH1-R	CCACGACCTCCAAAGACGG
	qRT-AtRD29A-F	ACGTTTGCTCCAAGTGGTGA
	qRT-AtRD29A-R	CCTCCAACGTTATCGGGGTC
	qRT-AtRD29B-F	GGCGGGCAAAGCGAG
	qRT-AtRD29B-R	TGCCCGTAAGCAGTAACAGATC
	qRT-AtDREB2A-F	GACCTAAATGGCGACGATGT

qRT-AtDREB2A-R	GCGGATCAAAACCACTTTGT
qRT-AtDi19-F	TTCGATCCCGATCTGAAATGT
qRT-AtDi19-R	TCTATGTGACAGCACAACCC
qRT-GhDi19-F	ATGGATGCTGATTCATGGAGT
qRT-GhDi19-R	TTATAAAATTTCATCAGGCAT
qRT-GhABA1-F	AAATCGTTTGTGATCGGAAGT
qRT-GhABA1-R	TAGCAACATTTGAAGGTATCCA
qRT-GhABA2-F	GCTCATTGCTTCCAGTCTCCA
qRT-GhABA2-R	TTTTCAGTCAACCCTACCACAGC
qRT-GhAAO3-F	GCAAACCCAAACTACACCCACA
qRT-GhAAO3-R	GCCACAGCCTCCTTCACCA
qRT- GhCDPK1-F	GGAGCCCATACTATGTTGC
qRT- GhCDPK1-R	GCCTTGCTCGCTTTCAG
